# Inhibition of ferroptosis-related NCF2 blocks the progression of lupus nephritis by activating PPARα pathway

**DOI:** 10.1186/s41065-025-00547-9

**Published:** 2025-10-01

**Authors:** Shiling Zhong, Yunyan Li, Yuanling Chen, Wei Jiang, Jika Zheng, Ling Wu

**Affiliations:** Department of Pediatric Rheumatology and Immunology, Women and Children’s Hospital of Ningbo University, No. 339 Liuting Street, Haishu District, Ningbo, 315000 Zhejiang China

**Keywords:** Lupus nephritis, NCF2, Ferroptosis, PPARα

## Abstract

**Background:**

Ferroptosis is involved in the pathogenesis of Lupus nephritis (LN), but its mechanism of action in LN remains unknown. This study aims to explore the effect of the ferroptositic-related gene neutrophil cytosolic factor 2 (NCF2) on LN and its potential downstream mechanism.

**Method:**

Differentially expressed genes (DEGs) between LN tissues and control tissues were screened out using “limma” R package. Weighted gene co-expression network analysis (WGCNA) was used to identify the key modules related to inflammation in LN based on DEGs. The genes associated with ferroptosis were obtained from the FerrDb database. Support vector machine recursive feature elimination (SVM-RFE) was used to screen candidate key genes. The expression and the diagnostic ability of candidate key genes was evaluated using an external validation set. Immune infiltration analysis was performed using CIBERSORT. Gene set enrichment analysis was used to reveal the molecular mechanisms of key genes. A cell model of LN was constructed using lipopolysaccharide (LPS) -induced human renal cortical proximal tubule epithelial cells HK-2 to explore the potential functions and mechanisms of the key gene NCF2 in LN.

**Result:**

Nine ferroptosis-related genes in LN were obtained after cross-analysis, and six candidate genes were screened out using machine learning approach. Among them, NCF2 was identified as a key gene related to ferroptosis in LN. The expression of NCF2 was positively correlated with the infiltration levels of pro-inflammatory cells such as monocytes and M1 macrophages, and negatively correlated with those of anti-inflammatory cells such as regulatory T cells (Tregs). NCF2-related DEGs were significantly enriched in the peroxisome proliferator-activated receptor (PPAR) signaling pathway. In vitro experiments demonstrated that knocking down NCF2 significantly inhibited LPS-induced suppression of viability, apoptosis, inflammatory response and ferroptosis of HK-2 cells. NCF2 knockdown also inhibited ferroptosis by activating the PPARα pathway.

**Conclusion:**

NCF2 is a key regulatory factor of LN. Its knockdown inhibits ferroptosis by activating the PPARα signaling, thereby alleviating inflammatory injury of renal tubular epithelial cells. Targeting NCF2 may provide a new strategy for the treatment of LN.

**Supplementary Information:**

The online version contains supplementary material available at 10.1186/s41065-025-00547-9.

## Introduction

Systemic lupus erythematosus (SLE) is a chronic autoimmune disease characterized by inflammation and immune-mediated damage to multiple organs. Approximately 3.4 million people worldwide have been diagnosed with SLE [1]. The kidney is the organ frequently affected in patients with SLE. Approximately 45–85% of patients present with clinical symptoms of lupus nephritis (LN) [2]. LN usually leads to irreversible kidney damage, and up to 20% of cases inevitably develop into end-stage renal disease (ESRD) [3, 4]. LN is usually treated with immunosuppressive drugs, such as glucocorticoids and cyclophosphamide or mycophenolate mofetil [5]. Unfortunately, these treatments are not effective for all patients and are accompanied by side effects [6, 7]. Therefore, in-depth research on the potential pathogenesis of LN is particularly important for identifying reliable biomarkers and effective therapeutic targets.

Ferroptosis is an iron-dependent, lipid peroxidization-driven cascade of cell death. It is different from other forms of cell death such as apoptosis, necrosis and autophagy. Its main characteristics are the production of a large amount of reactive oxygen species (ROS), iron-based lipid peroxidation and oxide accumulation [8, 9]. Ferroptosis is associated with a variety of diseases, including LN [10–13]. However, the ferroptosis mechanism in LN has not been fully elucidated at present. Neutrophil cytosolic factor 2 (NCF2) is a component of the NADPH oxidase complex in leukocytes that produces superoxides, participates in the generation of ROS, and is closely related to oxidative stress, autophagy, apoptosis and ferroptosis [14]. Some studies have reported that NCF2 plays a key role in the development of autoimmune diseases, such as rheumatoid arthritis, inflammatory bowel disease and SLE [15–17]. Notably, it is reported that NCF2 is highly expressed in the urine of LN mice and can serve as a key ferroptosis regulator in the glomerulus and tubulointerstitium of LN [11]. However, the mechanism of action of NCF2 in LN remains unclear at present.

In this study, the transcriptome data related to LN were analyzed. Next, key ferroptosis regulators related to LN were screened through machine learning algorithms, and the diagnostic potential of these genes for LN was evaluated using the receiver operating characteristic (ROC) curve. Finally, the function and molecular mechanism of the key gene NCF2 in LN were verified through in vitro experiments, and its influence on ferroptosis was explored. This study reports that NCF2 is a key diagnostic indicator and therapeutic target for LN. Its knockdown can inhibit ferroptosis by activating the peroxisome proliferator-activated receptor α (PPARα) signaling pathway, thereby blocking the progression of LN.

## Materials and methods

### Data download and preprocessing

With “lupus nephritis” and “homo sapiens” as the keywords, LN-related datasets were obtained from the GEO database (http://www.ncbi.nlm.nih.gov/geo). This study collected two datasets GSE32591 and GSE81622. GSE32591 (platform: GPL14663) contains 64 LN samples and 29 control samples, serving as the test set. GSE81622 (platform: GPL10558) contains 15 LN samples and 25 control samples, as an external validation set. The data was standardized using the R package affy, and the gene symbols were annotated using the corresponding platform annotation files.

### Identification of differentially expressed genes (DEGs) in LN

Difference expression analysis was conducted using the classical Bayesian and linear regression methods provided in the “limma” R package to identify DEGs between LN and the control samples. The screening criterion for DEGs was set to adj.P.Val < 0.05. The volcano map was constructed using fold change values and *P* values.

### Inflammatory response score

Single-sample gene set enrichment analysis (ssGSEA) is an improvement of gene set enrichment analysis, which generates the enrichment score of a given gene set for a single sample [18]. A total of 737 inflammatory response genes were obtained from the Gene Ontology (GO) item “inflammatory response” (GO: 0006954). The inflammatory response scores of all samples were calculated using the “GSVA” R package.

### Weighted gene co-expression network analysis (WGCNA)

Based on the expression profile data of all DEGs in the LN samples of the GSE32591 dataset, the gene co-expression network was constructed using the “WGCNA” R software package. Based on the principle of scale-free networks, a scale-free co-expression network was gradually established through a soft threshold (β), and the adjacency matrix was transformed into a topological overlap matrix. The hierarchical clustering tree of genes was generated through hierarchical clustering, and the dynamic tree cutting method was adopted to identify the highly correlated gene co-expression modules. The minimum number of genes in each gene module was set to 50. Then, the Pearson correlation test was used to analyze the correlation between the module eigengene (ME) and the inflammatory response score. When *P* < 0.05, it was considered that this module was significantly correlated with the Inflammatory response score. The key module was defined as the one with the highest correlation coefficient with the immune inflammatory response score, and the genes in this module were defined as key module genes.

### Identification and functional enrichment analysis of LN-related ferroptosis genes

Ferroptosis related genes including drivers, suppressors, markers, unclassified genes were obtained from FerrDb database (http://www.zhounan.org/ferrdb/). After merging and deleting the duplicate genes, 487 ferroptosia-related genes were obtained. Cross-analysis was conducted between the genes in the key modules recognized by WGCNA and the genes related to ferroptosis. Then, the “clusterProfiler” R package was used to conduct GO and Kyoto Encyclopedia of Genes and Genomes (KEGG) pathway analyses.

### Screening and verification of key genes

The feature gene screening was carried out by using the support vector machine-recursive feature elimination (SVM-RFE) algorithm. Based on the expression of LN-related ferroptosis genes in all samples, SVM-RFE was performed using the “e1071” R package, and the optimal number of features was selected through ten-fold cross-validation. Finally, the gene features in the optimal model were ranked by weight, and the top 6 genes were selected as candidate key genes. Based on the GSE32591 and GSE81622 datasets (external validation sets), the expression levels of the six candidate key genes were evaluated. Significance analysis was performed using the Wilcoxon rank sum test, and the results were visualized using the “ggplot2” R package. In addition, the ROC curve was established using the pROC package in R, and the area under the ROC curve (AUC) value was calculated to examine the diagnostic efficacy of the candidate key genes.

### Analysis of immune infiltration

CIBERSORT was a gene-based deconvolution algorithm that uses the features of 547 marker genes to quantify the relative scores of 22 types of human immune cells [19]. In this study, CIBERSORT algorithm was applied to analyze the proportion distribution of immune cells between LN and control samples in GSE32591 dataset. The “ggplot2” R package was used for drawing box plots. In addition, the Spearman correlation test was used to evaluate the correlation between key genes and immune cells.

### Difference analysis and functional enrichment analysis based on NCF2

The median expression of NCF2 in LN patient samples was used dividing the samples into high-expression and low-expression groups, and differential expression analysis was conducted with “limma” package in R. The “clusterProfiler” package in R was used to conduct GO and KEGG pathway enrichment analysis on DEGs.

### Cell culture and processing

Human renal cortical proximal convoluted tubule epithelial cells (HK-2) were purchased from the Typical Culture Collection Center of China (Wuhan, China). The cells were cultured in DMEM/F12 medium (Invitrogen, Carlsbad, CA, USA) containing 10% fetal bovine serum (FBS) (Thermo Fisher Scientific, Waltham, MA, USA) and 1% antibiotics (penicillin/streptomycin) (Invitrogen, Carlsbad, CA, USA) and maintained in an incubator at 37 °C containing 5% CO_2_. Referring to previous studies [11, 20], lipopolysaccharide (LPS) was used to treat HK-2 cells for 12 h as the cell model. The same volume of phosphate buffered saline (PBS) was used as the control. Small interfering RNA negative control (si-NC) and three siRNAs targeting NCF2 (si-NCF2#1, si-NCF2#2 and si-NCF2#3) were purchased from GenePharma Co.,Ltd. (Shanghai, China). Cell transfection was performed using Lipofectamine™ 3000 (Thermo Fisher Scientific Inc., Waltham, MA, USA). To explore the relationship between NCF2 and ferroptosis or the PPARα pathway, NCF2 knockdown model was constructed after LPS treatment (5 µg/mL, 12 h), followed by the ferroptosis inducer Erastin treatment (5 µM; MedChemExpress, Shanghai, China) or PPARα antagonist GW6471 treatment (10 µM; MedChemExpress, Shanghai, China) for 24 h.

### Cell counting kit-8 (CCK-8) assay

HK-2 cells were seeded in 96-well plates at a density of 5 × 10^3^ cells per well and cultured overnight. Subsequently, 10 µL of CCK-8 solution (Beyotime, Shanghai, China) was added to each well and incubated at 37 ° C for 2 h. The absorbance (optical density, OD) values of each well were detected at a wavelength of 450 nm using a microplate reader (Bio-Rad, Hercules, CA, USA). Cell viability (%) = (OD_test_ - OD_blank_)/(OD_control_ - OD_blank_) ×100%.

### Real-time quantitative polymerase chain reaction (RT-qPCR)

2 µg of RNA was reverse transcribed into complementary DNA (cDNA) using the PrimeScript™RT kit (Takara, Dalian, China). RT-qPCR was then perfromed using 2×SYBR Green RT-qPCR Master Mix kit (Selleckchem, Houston, TX, USA) in the CFX Connect real-time PCR detection system (Bio-Rad, Hercules, CA, USA). The relative expression of the target gene was calculated using the 2^−△△CT^ method, and the relative expression level of NCF2 was normalized with GAPDH. The primer sequences used in this study are as follows: NCF2 forward 5’-GGAGTGTGTCTGGAAGCAGAA-3’ and reverse 5’-TCTGCCCGTTGAACATGACC-3’; GAPDH forward 5’-CCACTAGGCGCTCACTGTTC-3’ and reverse 5’-TGGTTCACACCCATGACGAA-3’.

### Western blot assay

Equal amounts of protein (20 µg) of the samples were isolated using sodium dodecyl sulfate-polyacrylamide gel electrophoresis and transferred onto polyvinylidene fluoride (PVDF) membranes (Millipore, Billerica, MA, USA). Then the membranes were blocked with 5% skimmed milk at room temperature for 1 h, and then the membranes were blocked with the primary antibodies: anti-NCF2 antibody (ab109366, 1:1000), anti-glutathione peroxidase 4 (GPX4) antibody (ab125066, 1:1000), anti-solute carrier family 7 member 11 (SLC7A11, 1:1000) antibody (ab216876, 1:1000), anti-PPARα antibody (ab314112, 1:1000) and anti-GAPDH antibody (ab9485, 1:5000) at 4 °C overnight. Then the membrane was incubated with goat anti-rabbit IgG H&L (ab6721, 1:5000) at 37 °C for 1 h. Finally, the bands were developed using the BeyoECL kit (Beyotime, Shanghai, China), and quantitative analysis was performed using Image Lab™ software (Bio-Rad, Hercules, CA, USA). The antibodies used in this study were all purchased from Abcam (Shanghai, China).

### Apoptosis experiment

The processed HK-2 cells were rinsed twice with cold sterile PBS. For each sample, the cells were resuspended in 100 µL binding buffer, and then incubated in the dark with 5 µL Annexin V-FITC and 5 µL propidium iodide (PI) (Beyotime, Shanghai, China) for 30 min. After washing, the stained cells were analyzed in a FACScan flow cytometer, and the results were analyzed by FlowJo v.10 software.

### Enzyme-linked immunosorbent assay (ELISA)

The processed HK-2 cells were collected and centrifuged (1000×g, 10 min, 4℃), and then the supernatant was collected. According to the manufacturer’s procedures, the concentrations of tumor necrosis factor -α (TNF-α), interleukin-6 (IL-6), and interleukin-1 β (IL-1β) in the supernatant were detected using the corresponding ELISA kits (Beyotime, Shanghai, China), respectively.

### Detection of reactive oxygen species (ROS)

The intracellular ROS levels were detected using the ROS detection kit (Beyotime, Shanghai, China). 2’, 7’ -dichlorodihydrofluorescein diacetate (DCFH-DA) was diluted with serum-free medium at a ratio of 1:1000. The treated HK-2 cells were washed with PBS. The cells were incubated in the dark with 10 µM DCFH-DA at 37 °C for 20 min, and then washed three times with serum-free medium. The fluorescence intensity was detected at the excitation wavelength of 488 nm and the emission wavelength of 525 nm using an microplate reader (Bio-Rad, Hercules, CA, USA).

### Detection of malondialdehyde (MDA), glutathione (GSH) and catalase (CAT)

The HK-2 cells in different groups were harvested and washed with PBS. The cells were incubated with RIPA lysis buffer (Beyotime, Shanghai, China), and the cell supernatant was collected after centrifugation. According to the manufacturer’s instructions, the levels of MDA and GSH were detected respectively using the corresponding MDA, GSH and CAT assay kits (KeyGEN Biotech, Nanjing, China).

### Determination of iron content

The processed HK-2 cells were collected and the level of ferrous ions (Fe²⁺) in the cells was detected using the Iron Content Assay Kit (Jiancheng, Nanjing, China). The samples were washed three times with PBS, homogenized, then incubated with iron-loaded buffer, and finally stained with the iron probe in the dark for 1 h. Finally, the level of Fe²⁺ was determined at a wavelength of 593 nm using an microplate reader.

### Statistical analysis

All experiments were conducted independently in triplicate. All data are expressed as mean ± standard deviation (SD). The data were statistically analyzed using SPSS 21.0 software (IBM Corp., Armonk, NY, USA). Intergroup comparisons were conducted using Student’s t-test, or one-way analysis of variance (ANOVA) with Tukey post-hoc test. A *P* value < 0.05 was considered statistically significant.

## Result

### Identification of the genes related to ferroptosis in LN

**Fig. 1:**
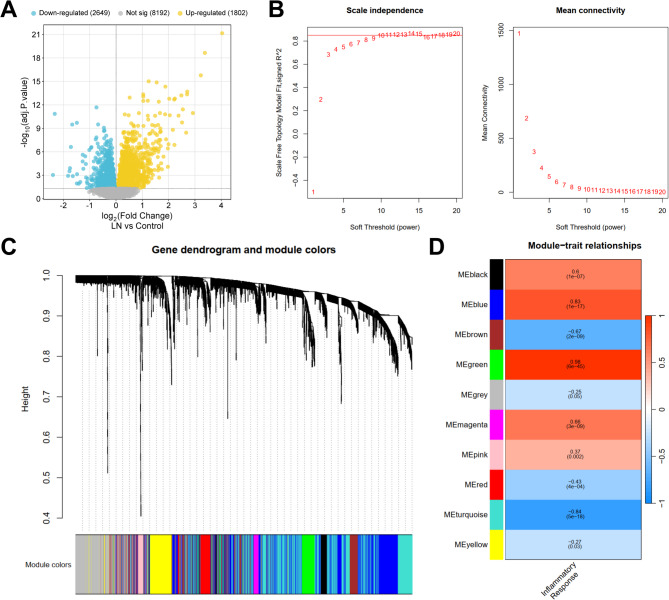
Construction and module analysis of weighted gene co-expression network analysis (WGCNA). **A**. The volcano plot shows the differentially expressed genes (DEGs) between the LN sample and the control sample. Blue dots represent down-regulated genes, yellow dots represent up-regulated genes, and gray dots represent genes with no significant difference. **B**. Network topology analysis under various soft threshold powers. Left: The X-axis represents the soft threshold power. The Y-axis represents the fitting index of the scale-free topological model. Right: The X-axis represents the soft threshold power. The Y-axis reflects the average connectivity. **C**. Gene hierarchical tree clustering graph. Different colors represent different modules. **D**. The heat map of the correlation between the gene module and the inflammatory response score. Each line corresponds to a gene module. Each box contains the corresponding correlation coefficient and *p*-value. Red indicates a positive correlation, while blue indicates a negative correlation. The darker the color, the stronger the correlation

**Fig. 2:**
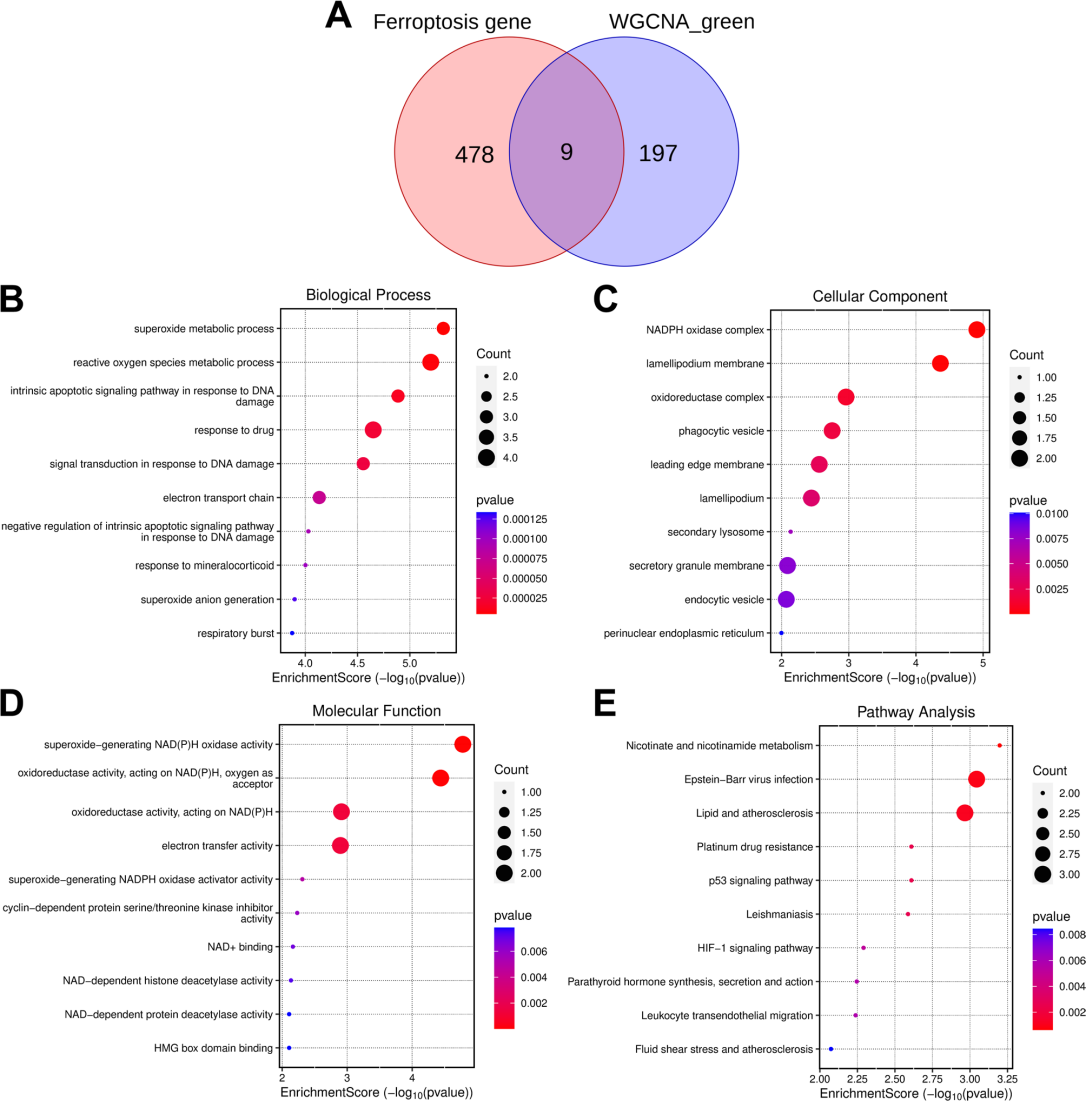
Identification and functional enrichment analysis of LN-related ferroptosis genes. **A**. The Venn diagram shows the intersection of the genes in key module (green module) and the genes related to ferroptosis. B-E. Bubble charts of the GO biological process (**B**), GO cellular components (**C**), GO molecular functions (**D**), and the results of KEGG pathway enrichment analysis (**E**) of LN-related ferroptosis genes. The color of the bubbles represents the *P* value, and the size of the bubbles represents the gene count

According to the screening criterion of adj.P.value < 0.05, a total of 4,451 DEGs in LN samples were identified, among which 2,649 genes were down-regulated and 1,802 genes were up-regulated (Fig. 1A). Subsequently, based on the expression profile data of DEGs in all LN samples, a gene co-expression network was established. The optimal soft threshold was set to 11 to construct the scale-free network with scale-free R^2^ values greater than 0.85 (Fig. 1B). Next, a gene hierarchical clustering tree diagram was constructed through gene correlation, and a total of 10 gene modules were identified (Fig. 1C). To identify the gene modules closely related to the trait of LN, inflammatory response score of LN samples was used as the trait, and pearson correlation analysis was performed between each gene module and the trait. The results showed that the green gene module was strongly positively correlated with the inflammatory response score of LN samples (*r* = 0.98, *p* = 6e-45) (Fig. 1D). Therefore, the green module was identified as a key module related to LN, and 206 genes in the module were identified as key module genes. Ferroptosis-related genes were collected from FerrDb database (http://www.zhounan.org/ferrdb/current/). Nine LN ferroptositic-related genes were obtained by intersecting the genes in the green module with those related to ferroptosis (Fig. 2A). GO analysis indicated that these genes were enriched in 425 biological processes, 29 cellular components and 35 molecular functions, mainly about oxidative stress (Fig. 2B-D). KEGG pathway enrichment analysis indicated that the 9 LN-related ferroptosis genes were significantly enriched in pathways/biological events such as “nicotinate and nicotinamide metabolism”, “p53 signaling pathway”, and “HIF-1 signaling pathway” (Fig. 2E).

### Identification and verification of key genes in modulating ferroptosis in LN

**Fig. 3:**
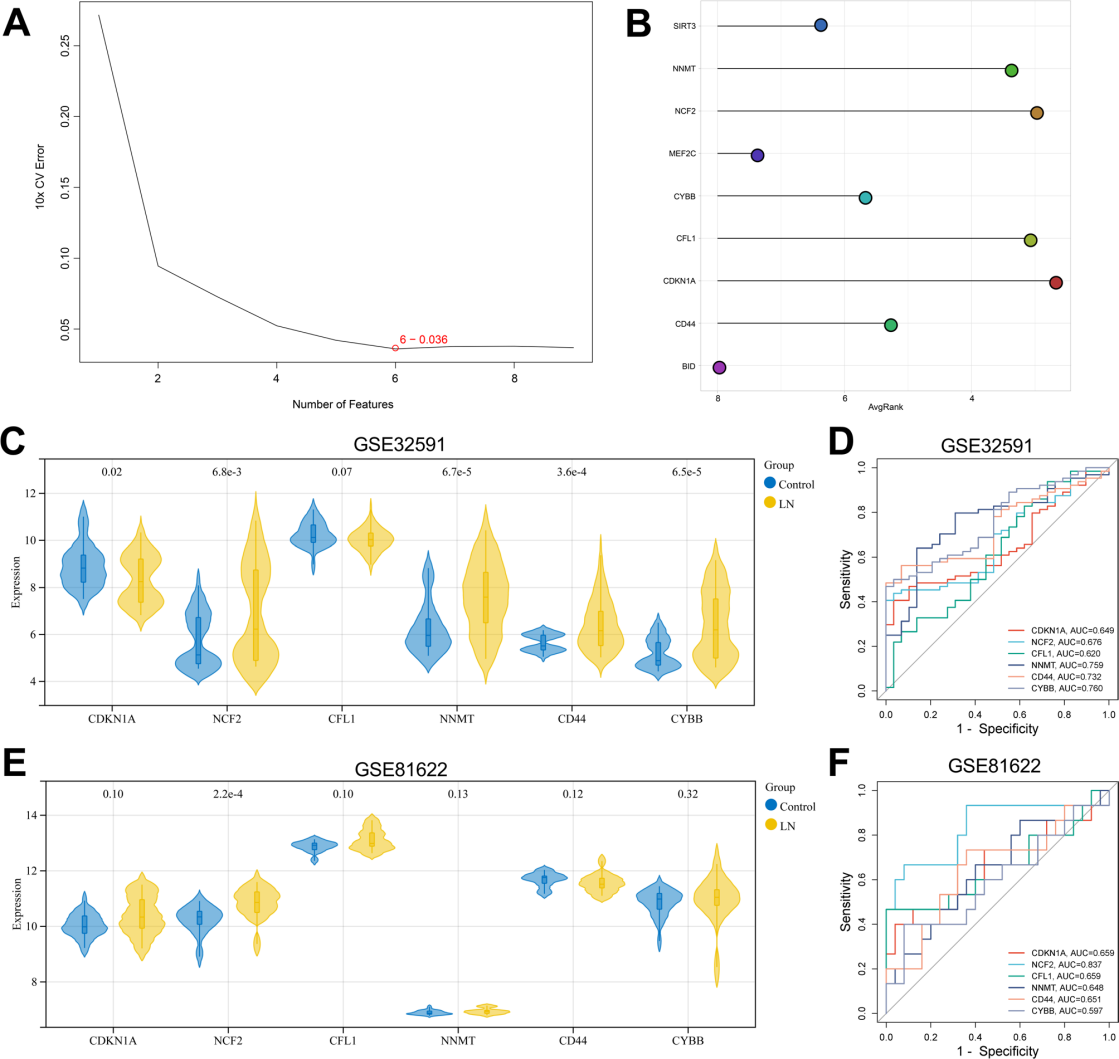
Identification and verification of key genes in regulating ferroptosis in LN. **A**: The minimum error plot of the SVM-RFE algorithm used for screening key genes. **B**. The bar chart shows the weights of candidate genes in the optimal model. **C**. Expression levels of six candidate key genes in LN samples and control samples of the GSE32591 dataset. **D**. ROC curves of six candidate key genes in the GSE32591 dataset. **E**. Expression levels of six candidate key genes in LN samples and control samples of the GSE81622 dataset. **F**. ROC curves of six candidate key genes in the GSE81622 dataset

**Fig. 4:**
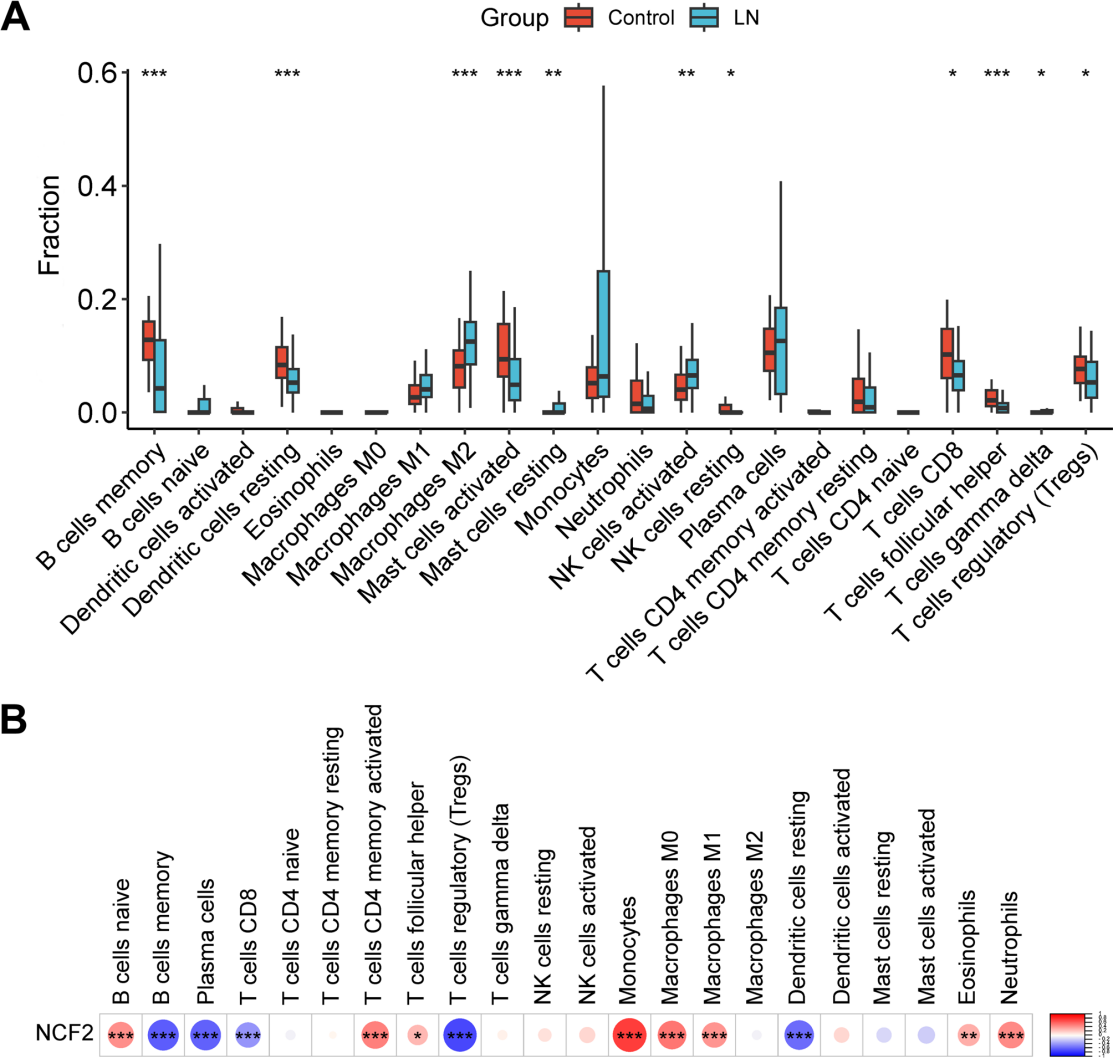
Analysis of immune cell infiltration. **A**. The box plot shows the infiltration differences of 22 immune cell subsets between LN samples and control samples. The color code of the LN group is blue, and that of the control group is red. **B**. Spearman correlation between the expression level of NCF2 and the abundance of 22 types of immune cells. Red indicates a positive correlation and blue indicates a negative correlation. *P < 0.05, **P < 0.01, **P < 0.001

To further identify the key genes involved in regulating ferroptosis in LN, SVM-RFE was adopted, and the optimal number of features was selected through ten-fold cross-validation (Fig. 3A). When the model error was the smallest, it contained six gene features. Subsequently, the gene features in the optimal model were weighted and ranked, and the top 6 genes were screened out, which were CDKN1A, NCF2, CFL1, NNMT, CD44 and CYBB (Fig. 3B). Based on the GSE32591 dataset, the expression levels of six candidate key genes in LN samples and the control samples were compared. Compared with the control samples, the expression of CDKN1A in LN samples was significantly decreased, while the expressions of NCF2, NNMT, CD44 and CYBB were significantly increased, while the expression of CFL1 showed no significant difference (Fig. 3C). ROC curve analysis indicated that the AUC values of the six genes in the GSE32591 dataset were all greater than 0.6 (ranging from 0.620 to 0.760) (Fig. 3D). GSE81622 was used as an external validation set to identify their expression levels and diagnostic value. The results showed that the expression of NCF2 in LN was significantly higher than that in the control group, while there was no significant difference in the expression of CDKN1A, CFL1, NNMT, CD44, and CYBB between the LN group and the control group (Fig. 3E). ROC curve analysis indicated that the AUC value of NCF2 was 0.837, while the AUC values of CDKN1A, CFL1, NNMT, CD44, and CYBB were all less than 0.7 (ranging from 0.597 to 0.659) (Fig. 3F). Therefore, NCF2 was chosen as a key gene, probably in regulating ferroptosis in LN, for subsequent research. As the pathogenesis of LN is closely related to the continuous renal inflammatory response mediated by immune cells, CIBERSORT algorithm was used to evaluate the differences in immune cell infiltration between the LN group and the control group. Compared with the control sample, in the LN group, B cells memory, Dendritic cells resting, Mast cells activated, NK cells resting, T cells CD8, T cells follicular helper, T The infiltration abundance of cells regulatory (Tregs) was relatively low, while that of macrophages M2, mast cells resting, NK cells activated, and T cells gamma delta was relatively high (Fig. 4A). Spearman correlation analysis indicated that the expression level of NCF2 was positively correlated with that of B cells naive (cor = 0.439, *p* = 1.05e-05) and T cells CD4 memory activated (cor = 0.489, *p* = 6.64e-07), T cells follicular helper (cor = 0.260, *p* = 0.012), Monocytes (cor = 0.742, *p* = 1.75e-17), macrophages M0 (cor = 0.526, *p* = 6.19e-08), Macrophages M1 (or = 0.411, *p* = 4.27e-05), eosinophils (cor = 0.309, *p* = 0.003), and neutrophils (cor = 0.458, *p* = 3.96e-06); and it was negatively correlated with B cells memory (cor = -0.623, *p* = 2.70e-11), plasma cells (cor = -0.608, *p* = 1.04e-10), and T cells CD8 (cor = -0.410, *p* = 4.27e-05), T cells regulatory (Tregs) (cor = -0.719, *p* = 4.74e-16), dendritic cells resting (cor = -0.5635, *p* = 4.40e-09) (Fig. 4B).

### Functional analysis of NCF2

**Fig. 5:**
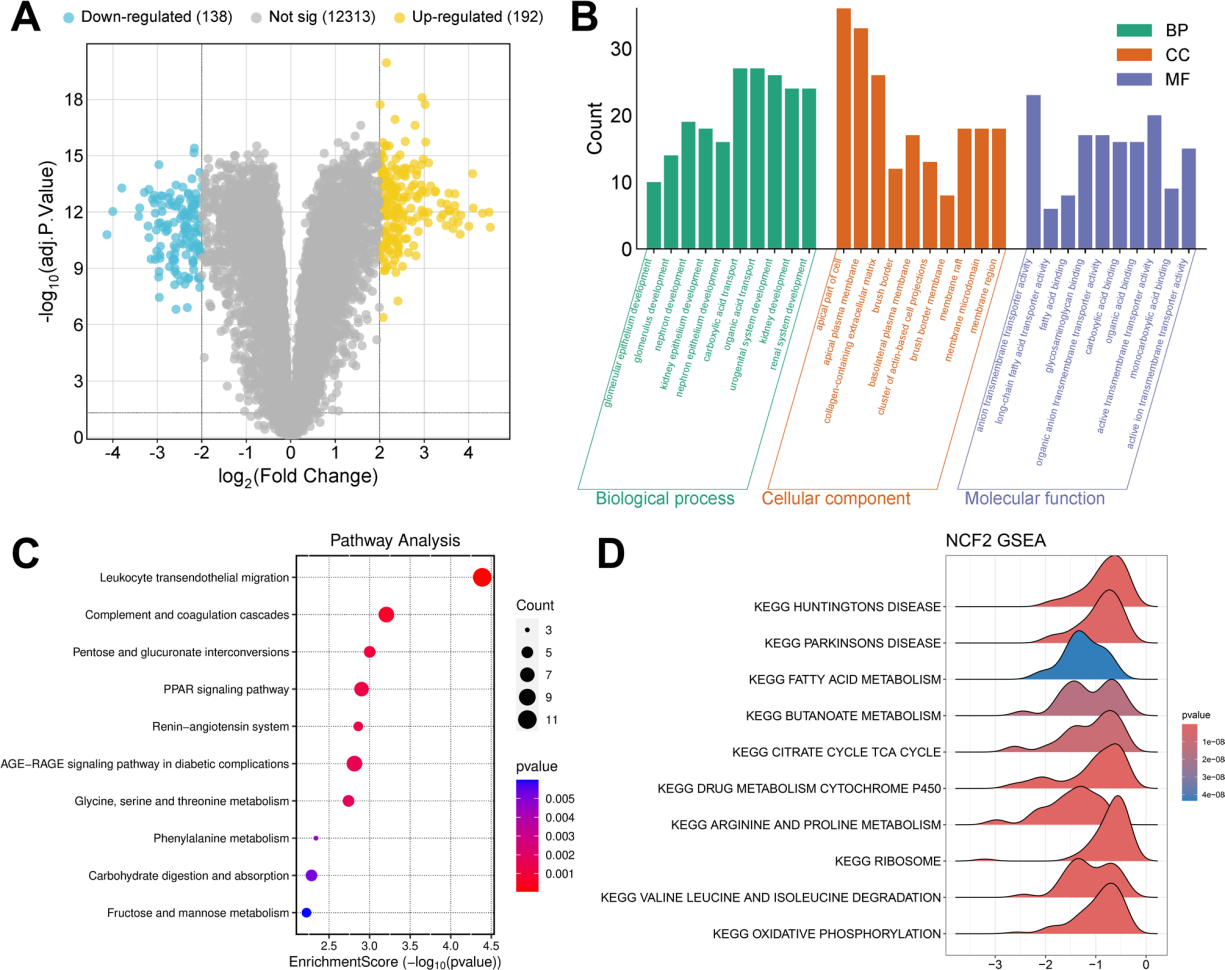
*In silico* functional analysis of NCF2. **A**. Volcano plot of the DEGs between the high-expression group and the low-expression group of NCF2 in GSE32591. Blue dots represent down-regulated genes, yellow dots represent up-regulated genes, and gray dots represent genes with no significant difference. **B**. The histogram of the results of GO analysis based on the DEGs. Biological process (BP) is marked by dark cyan, cellular component (CC) is marked by sienna and molecular function (MF) is marked by steel blue. **C**. The bubble plot of KEGG pathway enrichment analysis for the DEGs. The size of the bubbles represents count, and the color of the bubbles represents *P* value. **D**. GSEA of NCF2 based on the KEGG pathway gene set. The top 10 pathways with *P*.adjust < 0.05 were selected for visualization

According to the median expression value of NCF2, the LN samples in GSE32591 were divided into the high-expression group and the low-expression group. A total of 330 DEGs were identified between the two groups, among which 138 genes were down-regulated and 192 genes were up-regulated (Fig. 5A). To investigate the functional effects of NCF2, GO and KEGG pathway enrichment analyses were performed on the 330 DEGs using the “clusterProfiler” R package. GO analysis indicated that these DEGs were mainly involved in regulating glomerular epithelium development, nephron development, etc. (Fig. 5B). Furthermore, these 330 DEGs were significantly enriched in multiple pathways including PPAR signaling pathway, renin-angiotensin system, etc. (Fig. 5C). To further explore the potential molecular mechanism by which NCF2 participated in the progression of LN, a single-gene GSEA on NCF2 was performed. The results showed that NCF2 was negatively correlated with oxidative phosphorylation, valine leucine and isoleucine degradation, ribosome, huntingtons disease, drug metabolism cytochrome p450, arginine and proline metabolism, parkinsons disease, citrate cycle, tca cycle, butanoate metabolism and fatty acid metabolism (Fig. 5D).

### Knockdown of NCF2 alleviated LPS-induced injury of HK-2 cells

**Fig. 6:**
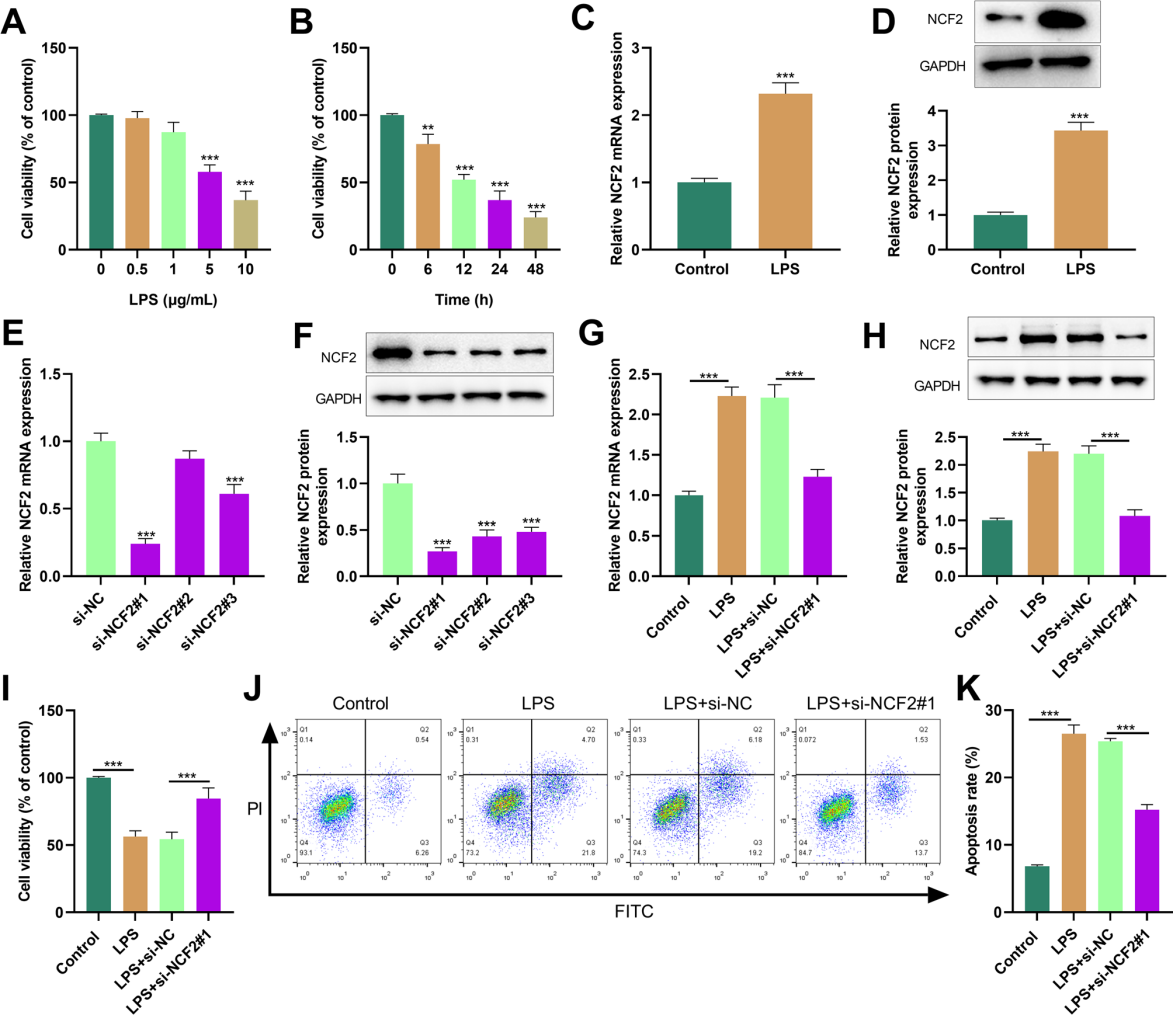
Effect of NCF2 knockdown on LPS-induced HK-2 cell injury. **A**. The survival rate of HK-2 cells after 12 h of LPS intervention at different concentrations (0, 0.5, 1, 5 and 10 µM) was detected by the CCK-8 method. **B**. The survival rate of HK-2 cells after treatment with 5 µM LPS for different durations (0, 6, 12, 24 and 48 h) was detected by the CCK-8 method. **C&D**. The mRNA and protein expression levels of NCF2 in HK-2 cells with or without 5 µM LPS treatment were detected by RT-qPCR (**C**) and Western blot (**D**). E&F. The mRNA and protein expressions of NCF2 in HK-2 cells transfected with si-NCF2 and its control were detected by RT-qPCR (**C**) and Western blot (**D**). **G&H**. HK-2 cells were transfected with si-NCF2#1 and its control for 48 h, and then stimulated with 5 µM LPS for 12 h. The mRNA and protein expressions of NCF2 were detected by RT-qPCR (**G**) and Western blot (**H**). **I**. Cell survival rate was detected by the CCK-8 method (**I**). **J&K**. Flow cytometry was used to analyze the cell death of HK-2 cells. The data are expressed as “mean ± SD”, with *n* = 3. **P < 0.01, ***P < 0.001

The CCK-8 assay showed that when HK-2 cells were treated with 5 µg/mL LPS, the cell survival rate was approximately 58% (Fig. 6A); when HK-2 cells were treated with 5 µg/mL LPS for 12 h, the cell survival rate was approximately 52% (Fig. 6B). Therefore, for the subsequent experiments, a concentration of 5 µg/mL and a treatment time of 12 h were selected as the standard conditions. LPS intervention significantly reduced the mRNA and protein expression levels of NCF2 in HK-2 cells (Fig. 6C&D). Subsequently, we knocked down NCF2 in HK-2 cells by siRNAs (Fig. 6E&F). Since the knockdown efficiency of si-NCF1#1 was relatively significant, it was selected in the subsequent experiments. Compared with the LPS + si-NC group, the mRNA and protein expression levels of NCF2 in HK-2 cells of the LPS + si-NCF2#1 group were significantly decreased (Fig. 6G&H). The inhibitory effect of LPS on the viability of HK-2 cells was reversed by NCF2 knockdown (Fig. 6I). In addition, LPS treatment significantly increased the cell death rate of HK-2 cells, while NCF2 knockdown inhibited LPS-induced cell death (Fig. 6J&K).

### Knockdown of NCF2 inhibited LPS-induced ferroptosis in HK-2 cells and activated the PPARα signaling pathway

**Fig. 7:**
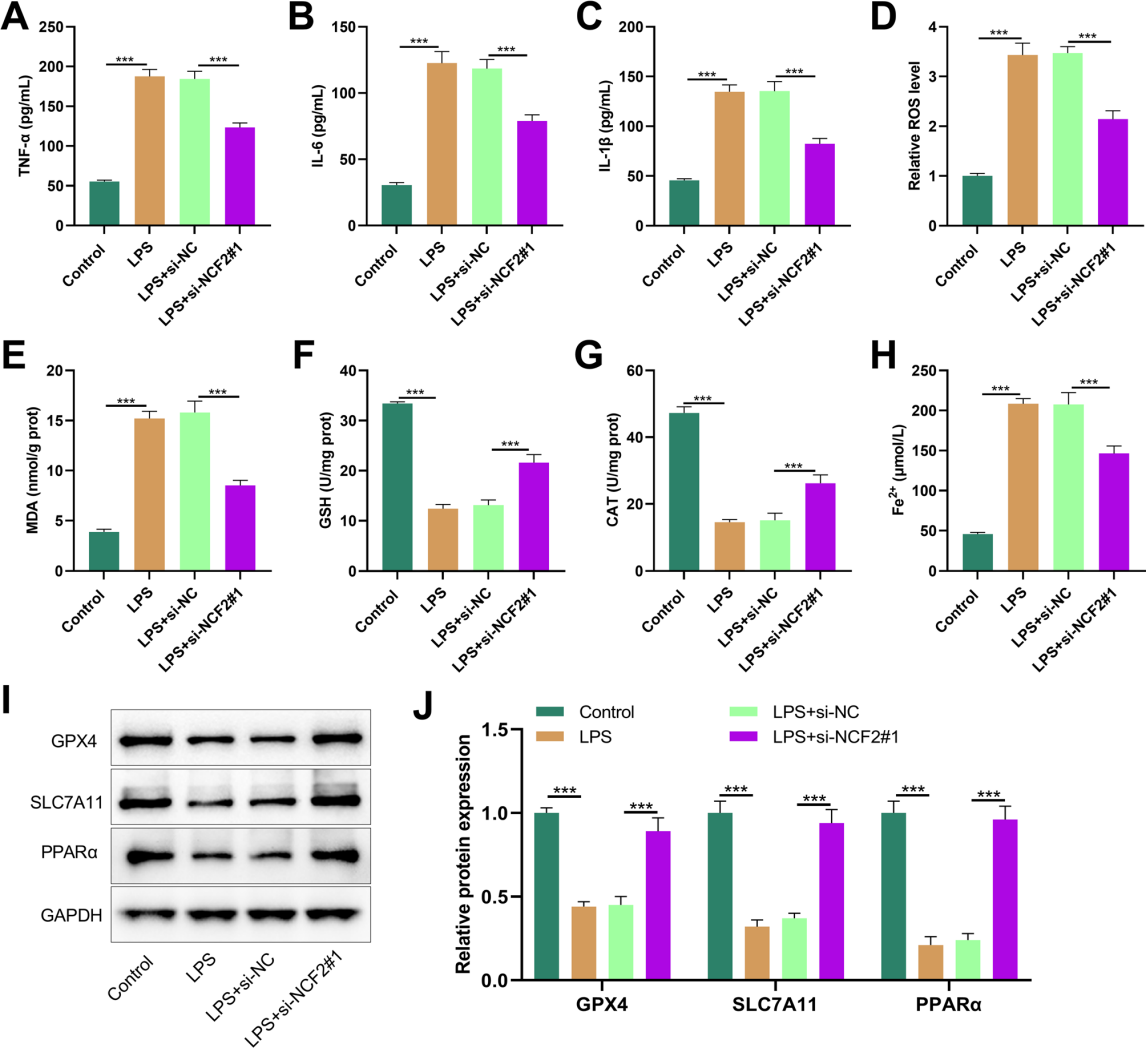
Effects of NCF2 knockdown on LPS-induced inflammatory response and ferroptosis in HK-2 cells. **A-C**. HK-2 cells were transfected with si-NCF2#1 and its control for 48 h, and then stimulated with 5 µM LPS for 12 h. The secretion of TNF-α (**A**), IL-1β (**B**) and IL-6 (**C**) in HK-2 cells was detected by ELISA. D. Detect the intracellular ROS level using DFCH-DA. **E-G**. The contents of MDA (**E**), GSH (**F**) and CAT (**G**) in HK-2 cells were determined respectively. **H**. Quantitative analysis of Fe^2+^ levels in HK-2 cells. **I&J**. Western blot was used to detect the protein expression levels of GPX4, SCL7A11 and PPARα in HK-2 cells. The data are expressed as “mean ± SD”, with *n* = 3. ***P < 0.001

Next, we evaluated the effect of NCF2 on the inflammatory response of LPS-induced HK-2 cells. ELISA showed that LPS stimulation significantly increased the levels of TNF-α, IL-1β and IL-6 in HK-2 cells, while the levels of these inflammatory factors significantly decreased after NCF2 knockdown (Fig. 7A-C). To determine whether NCF2 affected ferroptosis in LN, we examined the levels of ferroptosis markers such as ROS, MDA, GSH, CAT and Fe^2+^. LPS stimulation significantly increased the levels of ROS, MDA and Fe^2+^ in HK-2 cells, while reducing the contents of GSH and CAT (Fig. 7D-H). However, the depletion of NCF2 reversed these effects (Fig. 7D-H). In addition, as shown, LPS stimulation significantly inhibited the protein expression levels of GPX4 and SCL7A11 in HK-2 cells, while the protein expression levels of GPX4 and SCL7A11 were significantly increased after NCF2 knockdown (Fig. 7I&J). It is known that the activation of the PPARα signaling pathway can block the progression of LN [21]. As expected, the protein expression level of PPARα in LPS-treated HK-2 cells was significantly downregulated, while after NCF2 knockdown, it was significantly increased (Fig. 7I&J).

### NCF2 knockdown inhibits ferroptosis by activating the PPARα signaling pathway

**Fig. 8:**
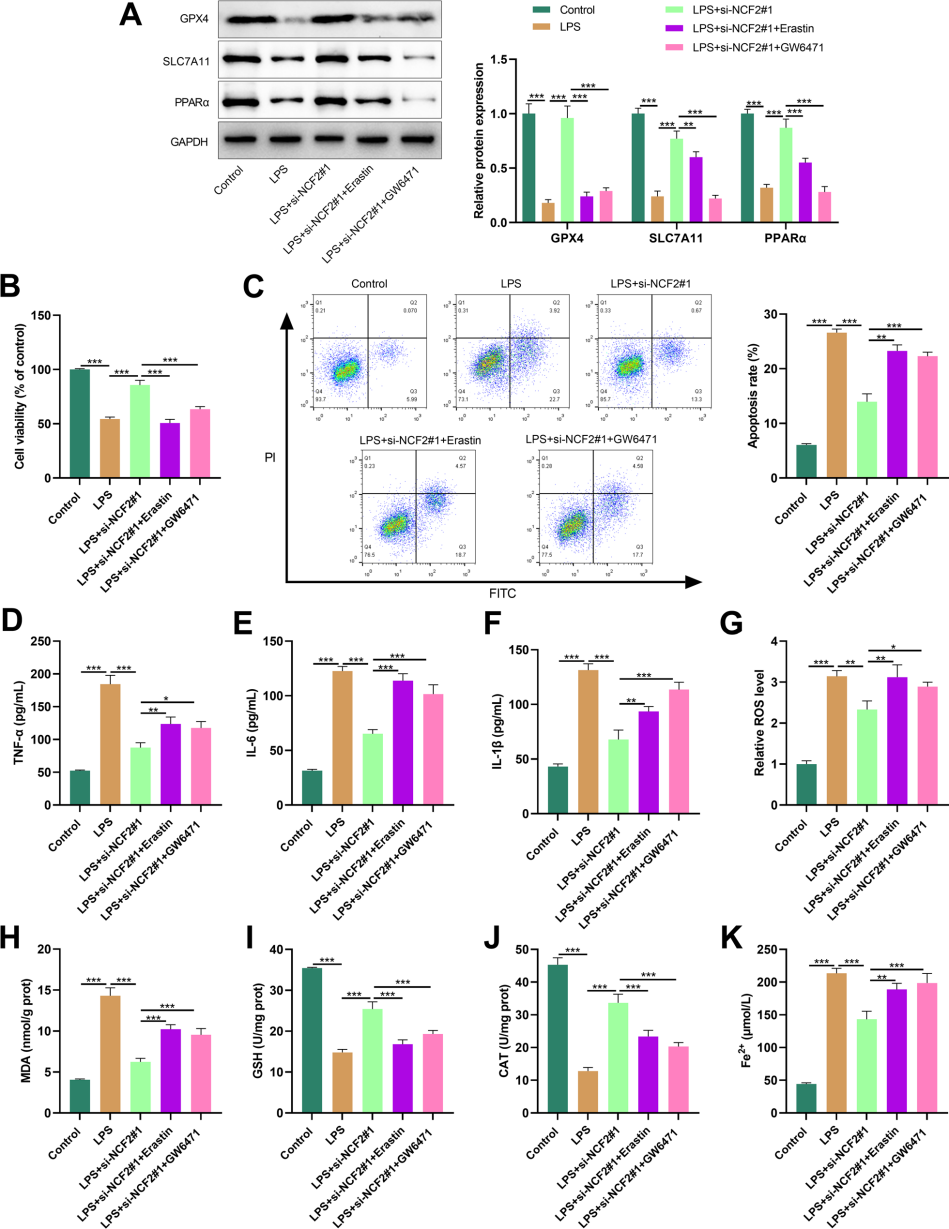
NCF2 knockdown inhibits LPS-induced ferroptosis in HK-2 cells by activating the PPARα signaling pathway. **A**. The HK-2 cells transfected with si-NCF2#1 were treated with LPS for 12 h and then incubated with Erastin or GW6471 for 24 h. The protein expression levels of GPX4, SCL7A11 and PPARα in HK-2 cells were detected by Western blot. **B**. Cell survival rate was detected by the CCK-8 method. **C**. Flow cytometry was used to analyze and evaluate apoptosis. **D-F**. The concentrations of TNF-α (**D**), IL-1β (**E**) and IL-6 (**F**) in HK-2 cells were detected by ELISA. G. Detect the intracellular ROS level using DFCH-DA. **H-J**. The contents of MDA (**H**), GSH (**I**) and CAT (**J**) in HK-2 cells were determined respectively. K. Quantitative analysis of Fe2 + levels in K.HK-2 cells. The data are expressed as “mean ± SD”, with *n* = 3. *P < 0.05, **P < 0.01, ***P < 0.001

To explore whether NCF2 is involved in the progression of LN by regulating the ferroptosis mechanism and the PPARα signaling, HK-2 cells with NCF2 knockdown were intervened with the ferroptosis inducer Erastin and the PPARα antagonist GW6471, respectively. Western blot showed that NCF2 knockdown significantly promoted the protein expression levels of GPX4, SCL7A11 and PPARα in LPS-induced HK-2 cells, while Erastin or GW6471 treatment partially reversed these promoting effects (Fig. 8A). NCF2 knockdown effectively alleviated LPS-induced inhibition of HK-2 cell viability, apoptosis and inflammatory response, but Erastin or GW6471 intervention could reverse the above protective effects (Fig. 8B-F). It was also found that compared with the LPS group, NCF2 knockdown significantly reduced the levels of ROS, MDA and Fe²⁺ in the cells, while increasing the contents of GSH and CAT; after treatment with Erastin or GW6471, the concentrations of ROS, MDA and Fe²⁺ significantly increased, while the contents of GSH and CAT significantly decreased (Fig. 8G-K).

## Discussion

LN is a kidney disease caused by SLE, and its pathogenesis involves complex immune inflammatory responses and imbalances in the regulation of cell death [22, 23]. Ferroptosis is a non-classical form of programmed cell death, characterized by iron overload, lipid peroxidation and oxidative stress [24]. An increasing amount of evidence has shown that ferroptosis plays a key role in the occurrence and progression of LN [25, 26]. It has been reported that the ferroptosis inhibitor ferrostatin-1 can alleviate podocyte injury in LN and renal injury in MRL/lpr mice [11]. This study, through the integration of bioinformatics analysis, machine learning and in vitro assays, for the first time, revealed that NCF2 is a key positive regulatory factor of ferroptosis in LN, and its knockdown inhibits ferroptosis by activating the PPARα signaling pathway, probably slowing down the progression of LN.

The NCF2 gene encodes the key subunit p67phox of the NADPH oxidase complex and plays a core role in ROS generation and immune regulation [27]. It is reported that the intron variant rs10911363 in the NCF2 gene is associated with SLE susceptibility in the European population [28]. NCF2 can serve as a biomarker related to ferroptosis in various diseases, such as Alzheimer’s disease [29], amyotrophic lateral sclerosis [30], sepsis-induced acute lung injury [31], and LN [11]. In this study, through bioinformatics analysis, 9 genes related to ferroptosis in LN were identified, and through machine learning, 6 key genes (CDKN1A, NCF2, CFL1, NNMT, CD44, CYBB) were screened out from them. Interestingly, in the independent validation of the external dataset GSE81622, only NCF2 was consistently and significantly highly expressed in the LN group and demonstrated excellent diagnostic efficacy (AUC value = 0.837), while the other genes lost their significance in cross-dataset validation. This result highlights the robustness of NCF2 as the core gene of LN. Immune cell infiltration is an important marker of LN [32, 33]. This study found that the expression level of NCF2 was significantly positively correlated with the infiltration abundance of various pro-inflammatory immune cells (such as monocytes, M1-type macrophages, and neutrophils), but negatively correlated with regulatory T cells (Tregs) with immunosuppressive functions. This suggests that NCF2 may exacerbate inflammatory injury in LN by reshaping the immune microenvironment. It has been reported that interfering with NCF2 can inhibit endothelial cell ferroptosis induced by oxidized low-density lipoprotein and alleviate arterial ferroptosis and inflammatory responses in atherosclerotic mice [14]. S100A8 can promote ferroptosis through the NCF2/NOX2 pathway, thereby promoting cyclophosphamide-induced alopecia [34]. In this study, we found that NCF2 was upregulated in LPS-treated HK-2 cells, which is consistent with previous reports [11]. Knockdown of NCF2 inhibited the suppression of viability, apoptosis, inflammatory response and ferroptosis of LPS-treated HK-2 cells. However, treatment with the ferroptosis inducer Erastin reversed the effect of NCF2 knockdown on LPS-induced inflammatory injury in HK-2 cells. These findings suggest that NCF2 can promote the progression of LN through the ferroptosis mechanism.

PPARα is an important member of the peroxisome proliferator-activated receptor (PPAR) family and functions as a nuclear transcription factor in fatty acid metabolism [35]. The PPARα pathway has been confirmed to be closely related to inflammation, oxidative stress, necrosis and apoptosis [36]. Importantly, activating the PPARα pathway can alleviate ferroptosis in the liver of mice induced by iron overload [37], and downregulation of PPARα induces ferroptosis in human mesangial cells by reducing the expression of the downstream target gene FABP1, thereby leading to immunoglobulin A nephropathy [38]. PPARα selective agonist WY14643 can improve LN by down-regulating the RORγT/STAT3 signaling pathway in MRL/lpr mice [21]. In this study, KEGG pathway enrichment analysis revealed that NCF2-related DEGs were significantly enriched in the PPAR signaling pathway. Consistently, in vitro experiments confirmed that NCF2 knockdown reduced the mRNA and protein expression levels of PPARα in LPS-induced HK-2 cells. However, when the PPARα antagonist GW6471 was used for intervention, the effect of NCF2 knockdown on ferroptosis was greatly reversed, with increased cell death and decreased viability. These results suggest that NCF2 may exacerbate ferroptosis in LN by inhibiting the PPARα pathway.

However, this study still has some limitations. Although the LPS-induced HK-2 cell model can simulate some inflammatory characteristics of LN, it cannot fully replicate key pathological processes such as immune complex deposition and podocyte injury in vivo. Future validation is required in Pristine induced lupus mouse models or primary renal cells of LN patients.

## Conclusion

This study, for the first time, confirms that NCF2 knockdown inhibits ferroptosis by activating the PPARα signaling pathway, thereby slowing down the progression of LN. This discovery not only enriches the understanding of the pathogenesis of LN, but also provides an important basis for the development of new targeted therapeutic strategies.

## Supplementary Information

Below is the link to the electronic supplementary material.

Supplementary Material 1

## Data Availability

The data used to support the findings of this study are available from the corresponding author upon request.
